# Development and Validation of a Set of Instruments to Measure Food Environments

**DOI:** 10.3390/ijerph192113806

**Published:** 2022-10-24

**Authors:** Jacqueline Araneda-Flores, Patricio Oliva Moresco, Gladys Quezada-Figueroa, Luz Lobos-Fernandez, Barbara Leyton, Anna Christina Pinheiro

**Affiliations:** 1 Departamento de Nutrición y Salud Pública, Facultad de Ciencias de la Salud y de los Alimentos, Universidad del Bío-Bío, Chillán 3780000, Chile; 2 Instituto de Nutrición y Tecnología de los Alimentos (INTA), Universidad de Chile, Santiago 8330015, Chile; 3 Carrera de Nutrición y Dietética, Facultad de Medicina-Clínica Alemana, Universidad del Desarrollo, Santiago 7550000, Chile

**Keywords:** food environment, Chile, nutrition, evaluation

## Abstract

Background: There is worldwide interest in measuring local food environments (FEs). The aim of this study was to develop and validate a set of instruments to evaluate FEs in Chile. Methods: Based on the development and validation of four instruments to measure FEs, a literature review, an evaluation by experts, and the implementation of a pilot tool in the FEs of schoolchildren from nine public schools in the commune of Chillán, Chile, were used. Results: A tool to evaluate FEs was provided, based on three dimensions: availability, variety, and advertising of healthy foods. A total of 1928 foods points of purchase were evaluated. The reliability was evaluated by Cronbach’s alpha. Some 74% of the foods’ points of purchase were store locations. The reliability of the four instruments was high to acceptable (store: 0.90; institution: 0.77; street food: 0.74; restaurant: 0.68). Unhealthy foods were highlighted by the scores obtained: store (6.08 ± 4.07; range: 0–13), restaurant (3.95 ± 1.75; range: 0–10), street food (1.18 ± 1.56; range: 0–7), and institution FEs (3.38 ± 2.78; range: 0–9). Conclusions: The results of this tool can provide information to governments for incorporating structural measures to ensure adequate availability, variety, and advertising of healthy foods in different FEs.

## 1. Introduction

One of the most serious problems worldwide in recent decades has been the increased prevalence of obesity and diet-related chronic diseases and premature death [1,2]. The interaction between factors related to food environments and social determinants of health plays an important role in this situation [3,4]. Over the last decade, an obesogenic environment has been established, which is characterized by a high supply of ultra-processed foods, low availability of healthy or natural foods, and scarce supply of safe and adequate settings for regular physical activity [5,6].

Food environments have been acknowledged as those environments where the physical presence or absence of food and beverages, proximity of food supply points, geographic distribution, and road networks that allow easy access to these foods points of purchase can affect the choices of food and beverages being consumed and, therefore, the nutritional status of individuals [7,8]. Five food environments have been identified in Chile, which determine food consumption in the country: (i) domestic/home: refers to the primary transmission of food preferences and reproduction of food practices in the home; (ii) street food: includes public areas, street vendors, and fast food; (iii) restaurant: considers fast food restaurants, take-out restaurants, “cocinerías” (small breakfast and lunch-only restaurants), bars, and hotels; (iv) institution: refers to kiosks, cafeterias, and vending machines inside schools, hospitals, and private and public companies; (v) stores: includes markets and farmer’s markets, supermarkets, produce stores, and bakeries [9].

An increasing number of studies has measured and explored the effects of food environments on the eating behavior of populations. Different methodologies such as the geographic location of food and beverage points of purchase, distance between the individual’s location (home, school, university, workplace, health centers, etc.), and the places where food is sold (exposure to supermarkets, markets, convenience stores, etc.) have been used to examine the distance and density of food points of purchase [10,11]. Another way to measure food environments is through the perception of individuals regarding the availability of healthy and unhealthy foods in their environment, culture, and food preferences [12,13,14].

International efforts have been reported on the development of tools that can measure food environments [15,16] to identify those areas with gaps in the accessibility (physical and economic) to healthier foods. These tools that are aimed at measuring food environments should accurately determine environmental exposure, including sensitive, specific, and appropriate measures to detect contexts and particularities, such as local regulations that could have an impact on the configuration of these environments.

The present report shows the development and validation process of instruments that consider the food environments identified by the Chilean Ministry of Health [9] and the food regulations in force in the country, such as the front-of-package (FOP) “high-in” label [17]. Therefore, the results provide a set of instruments to evaluate food environments based on three dimensions: availability, variety, and advertising of food in Chile.

## 2. Materials and Methods

The present study is part of project called “Exposure to food environments and diet quality in schoolchildren with obesity and normal weight in the Ñuble Region”. One of the objectives of this project was to construct instruments that would enable the measurement of food environments. This article describes two of the stages of this study: the development of Chile–food environments instruments and the implementation of the pilot instruments.

### 2.1. Development of Instruments

The instruments were developed during 2019. The first stage of construction included the planning and development process of four versions of instruments to measure the food environments identified by the Chilean Ministry of Health [9]: store, restaurant, institution, and street food. An iterative process was used to develop the first version of the instruments based on the existing literature. Our search included articles that described and applied tools to measure food environments in different countries and in varying contexts [15,16].

Three components were measured in each of the four instruments: (1) availability, (2) variety, and (3) heathy food advertising, including their respective domains and subdomains (Table 1). The availability component was constructed based on foods with higher apparent consumption, according to the 8^th^ Family Budget Survey conducted in Chile in 2018 (21). This survey grouped food in the following categories: fruit, vegetables, dairy products, legumes, meats and eggs, cereals, and beverages. In addition, in a final version of the instrument, a subitem was included for unhealthy foods consisting of ultra-processed products that have a front-of-package (FOP) “high-in” label [17]. In the case of street food, institution, and restaurant food environments, prepared foods usually consumed in Chile for breakfast, lunch, teatime, and dinner were added. The variety component considered the availability of two or more alternatives of foods and beverages or healthy prepared foods. The healthy advertising component considered questions related to exposure to healthy messages in the Chilean context, including food laws and recommendations issued by the Ministry of Health: (i) good-based dietary guidelines for the Chilean population (GABA) [18]; (ii) packaged foods with no FOP “high-in” labels [17]; (iii) reduced price of foods and/or menus regarded as healthy; (iv) food and menu promotions such as 2-for-1, food packs, or giveaways. Questions on the presence of promoters, food tastings, and positioning of food healthy products in prominent places in the supply food environments were also included. The evaluation of advertising did not consider advertising on public roads, such as billboards, signs on the streets, or at bus stops.

Content validation of the four instruments was performed by an advisory panel of experts in the field of food and nutrition, who were specifically selected to ensure input from different areas of the country (north, center, and south). In addition, leaders of trade local associations related to the commercialization of fruit and vegetables in Chile participated in this process. Two focus groups, with a total participation of 17 individuals, were designed and conducted by an anthropologist. The information was analyzed according to the grounded theory, and adjustments requested by the experts were made on the basis of this analysis. The three components of availability, variety, and advertising of food and beverages, as well as the refinement of each of the components, were included in each instrument (Supplementary Material Tables S1–S4). The instrument was designed to be used in any population group and geographic area as a dichotomic checklist (yes or no) with questions that scored 1 when the condition was met and 0 when the condition was not met. 

### 2.2. Implementation of Pilot Instruments

The final structure of the instruments and the comprehension of the questions were analyzed. The final versions of the four instruments were subsequently achieved and applied in a 5 mo pilot test in 2021 in the city of Chillán, located in southern Chile, to evaluate the feasibility of their implementation. The geographic area in which the instruments were applied included food environments within a radius of 400 m around the home and school attended by randomly selected (n = 253) schoolchildren from nine public schools. A total of 1928 foods points of purchase were evaluated by applying the respective instruments of store, restaurant, street food, and institution food environments (Table 2).

The information was collected by previously trained nutritionists who worked in pairs; the recorded responses were based on a consensus among the professionals. A 5 d training period was conducted via videoconference with a comprehensive review of the instruments; these instruments were applied in the designated territory (10 food outlets for each of the four types of food environments). The process of applying the instruments included walking along the streets of the city to directly observe the food points of purchase based on a radius of 400 m around the initially identified geographic points (home and school).

### 2.3. Statistical Analysis

The reliability of the instruments (internal consistency) was evaluated by Cronbach’s alpha [19], which varies between 0 and 1. The reliability coefficients were classified as high (0.8 to 1.0), acceptable (0.6 to 0.8), satisfactory (0.4 to 0.6), low (0.2 to 0.4), and very low (≤0.2).

The analysis of the evaluated dimensions (availability of healthy foods, availability of unhealthy foods, variety, and healthy food advertising) was based on positive responses (yes) with score of 1 and negative responses (no) with score of 0. Sum of scores were categorized according to the interquartile by considering their distribution range in each food environment. Scores were individually classified according to the availability, variety, and healthy food advertising components: (1) very low (less than 25th percentile); (2) low (between 25th and 50th percentiles); (3) intermediate (between 50th and 75th percentiles); (4) high (greater than 75th percentile). The statistical analyses were performed with Stata 16.1 software (College Station, TX, USA).

## 3. Results

Approximately 74.0% of the 1928 foods’ points of purchase in the city of Chillán were in the store category (Table 2). Table 3 displays the reliability analysis using Cronbach’s alpha for the four instruments. The highest degree of consistency was reached in the store instrument (0.90), while the institution and street food environments instruments had a value of 0.77. Finally, the restaurant instrument obtained a value of 0.68 (Table 3). This implies that the dimensions of the instruments are highly correlated with each other; that is, they measure the same concept.

Table 4 shows the total scores and subgroup scores for each dimension of the evaluated food environment. The possible score ranges for each subgroup are also shown. In the store food environment, the highest scores were obtained for dairy products (2.24 ± 2.14; range 0–6) and beverages (2.56 ± 1.77; range 0–5). The availability of unhealthy foods (6.08 ± 4.07; range: 0–13) was higher than that of healthy foods (10.62 ± 7.47; range: 0–28).

The highest scores for the restaurant food environment were for lunch (1.31 ± 1.33; range: 0–5) and dinner (1.09 ± 1.25; range: 0–6). The availability of unhealthy foods (3.95 ± 1.75; range: 0–10) was higher than that of healthy foods (3.41 ± 3.34; range: 0–22).

For the street food environment, the highest scores were for fruit (0.37 ± 0.54; range: 0–2), vegetables (0.36 ± 0.48; range: 0–1), and beverages (0.29 ± 0.81; range: 0–4). Scores for healthy (1.32 ± 1.28; range: 0–7) and unhealthy foods (1.18 ± 1.56; range: 0–7) were similar in this food environments.

The institution food environment had the highest scores for availability of fruit (0.88 ± 0.89; range: 0–3), beverages (2.00 ± 1.32: range: 0–4), and prepared foods (2.25 ± 2.86; range: 0–10). Unhealthy foods (3.38 ± 2.78; range: 0–9) had a higher score than healthy foods (4.88 ± 3.69; range: 0–14).

As for the variety of foods, the highest scores were for the store food environment (2.07 ± 2.10; range: 0–8). Meanwhile, the lowest scores were for the street food environment (0.36 ± 0.71; range: 0–3).

In healthy food advertising, the highest scores were obtained for the street food environment (0.74 ± 0.95; range: 0–2). The lowest scores were for the restaurant food environment (0.05 ± 0.26; range: 0–3).

## 4. Discussion

The identification and characterization of food environments requires the development and validation of tools to capture information in a local context, classify it, and, subsequently, address it as part of the public policies aimed at improving the availability of healthy and safe foods for the population. Our results made it possible to construct and validate four instruments that were able to identify and classify the food environments defined for Chile: store, street food, restaurant, and institution.

For a better understanding of the problem, different authors have indicated that the analysis of the causal factors of obesity implies the need to evaluate and analyze food environments [20,21]. The Food and Agriculture Organization of the United Nations (FAO) states that food environments determine when foods are affordable, desirable, and convenient to meet the needs of the population for a healthier life [22]. Therefore, the absence of foods considered to be healthy promotes food deserts, but when unhealthy foods outnumber healthy food options, food swamps occur. Both food deserts and swamps are associated with a higher prevalence of obesity and are predominantly found in more vulnerable territories [23,24].

Chile, currently, has one of the highest childhood obesity prevalence rates in the world [25]. Several public policies have been implemented in recent years, aimed at reducing the advance of obesity in the population [17,26]. Despite the significant regulatory advances and a series of campaigns aimed at promoting healthier diets, food consumption based on the dietary guidelines [18] has not evolved favorably. According to the national surveys that evaluate food consumption, less than 15% of the population meets the recommendations for the consumption of legumes and/or fish (twice a week); there is also a high consumption of foods with high concentrations of critical nutrients [27,28,29,30]. Given the serious obesity problem in the country, understanding its conditioning factors is essential for developing public policies that can efficiently and effectively address the issue. Therefore, identifying and analyzing food environments is considered as one of the most efficient strategies for evidence-based interventions, whereby the healthy option should be the easiest and most accessible [31].

Some proposed tools to measure food environments have been developed and published in recent years. Some of the most used instruments are the Nutrition Environment Measurement Surveys (NEMS), which consist of instruments designed to measure food environments in different settings (restaurants, commercial premises, etc.); these have been adapted for use in a number of countries [15,16,32]. Brazil has also proposed a tool to measure food environments, which is mainly focused on evaluating foods’ points of purchase that have sanitary authorization, such as supermarkets and grocery stores; it also considers the degree of food processing according to the NOVA classification [15].

The present Chilean proposal of instruments to evaluate food environments considers the local context and food environments previously identified by the Ministry of Health [9]. The development of these instruments considered the local regulations that identify those foods deemed to be more or less healthy, thus providing a better assessment of the population’s exposure to these products. The FOP “high-in” label on packaged foods was used to select healthier foods; the message of the Ministry of Health to the population is: “prefer to consume food products with fewer labels, and all the better if there are none”.

The development tool has also included aspects used in other studies for measuring food environments such as food availability, advertising, and variety of foods. It should be noted that the four proposed instruments showed an acceptable-to-high degree of internal consistency. Consistency was high in the store environment, related to settings such as supermarket and neighborhood stores. Moreover, results have indicated that the instruments were sensitive to environmental variations, which revealed the widespread availability of unhealthy versus healthy foods.

Various studies have demonstrated that the identification and classification of food environments could enable the adoption of targeted measures in environments where the most at-risk populations are found, such as the school environment. An increased supply of healthier foods in points of purchase inside (such as kiosks and vending machines) and outside (street vendors and local stores) schools can be good examples of actions based on the identification, classification, and subsequent modification of food environments [31]. In addition, this type of information is a valuable resource for local authorities to implement restrictive territorial measures, such as prohibiting the sale of unhealthy foods around or inside schools [17,33,34].

It should be noted that the theories that consider healthy food selection as an individual decision do not provide sufficient evidence to explain the causal factors of obesity. In relation to childhood obesity, the socio-ecological model postulates that healthy lifestyle choices of individuals are the result of a complex relationship between strongly interconnected systems, which can lead to the creation of unfavorable environments for the health of the most vulnerable population [30]. One of the gaps observed in the research, which considers the socio-ecological model to study the conditioning factors of childhood obesity, refers to the limited inclusion of the macrosystem in the analyses, given that the macrosystem includes the identification of food environments [35,36].

Our study is the first to prepare and validate a tool to measure food environments in Chile. Its dimensions (availability of healthy and unhealthy foods, variety, and healthy food advertising), to a greater or lesser extent, outline the characteristics of food environments in the area in which it was applied. The territorial use of this instrument based on different local situations, such as income level and population density, could be used to more clearly identify areas in need of greater or lesser intervention by local governments. Interventions could be through structural measures or direct actions with the population, such as promoting conditional cash transfer programs or increased number of food points of purchase of healthier foods.

Food availability in the school environment was evaluated with the instruments presented in this study. They classified the formal and informal food points of purchase found inside and 100 m around the schools. When relating the point of purchase classification to determine territorial sociodemographic characteristics, it was possible to demonstrate that territories with higher multidimensional poverty showed lower healthier food availability in the school environment [11].

Some authors have described the processes of quality indicator analysis of instruments at various stages [36], including reliability and validity. One of the limitations of the present study is that the results of applying the instrument were not compared with widely used instruments such as the NEMS [16]. Another limitation that was detected when developing the four instruments is that they did not consider the survey of prices at the point of purchase or how the food was used. Other authors have also suggested the identification of the quality and sustainability properties of foods in the evaluation of food environments [37]. In addition, information related to the nutritional composition of the foods and beverages observed in the different food environments was not included.

## 5. Conclusions

Findings showed that the instruments that were developed are a valid and reliable tool to evaluate the food environments in Chile. These could be adapted to different contexts and countries using their dimensions (availability, variety, and advertising of healthy foods), which show theoretical and methodological cohesuprence. This enables an objective description of the different types of food environments to which consumers have access in their daily lives and to the development and implementation of interventions and policies that promote the consumption of healthy and sustainable diets.

## Figures and Tables

**Table 1:** General description of components, domains, and subdomains of the instruments to measure food environments in Chile.

Component	Domains	Subdomains
Availability	Healthy foods	Fruit, vegetables, dairy products, legumes, meats and eggs, cereals, beverages, others
	Prepared foods	Soups, sandwiches, main dishes, dessert, infusions
	Unhealthy foods	Sweet and salty snacks, sausages and cold cuts, fast food, sweetened beverages, ice cream
Variety	Healthy foods	Fruit, vegetables, milk products and derivatives, low-fat meats, breakfast cereals without the “high-in” logo, water and unsweetened beverages, foods that are natural, fresh, frozen, canned, and/or in packs
Healthy food advertising	Promotion	Healthy food and beverage advertising
	Price	Promotional prices, strategies such as 2-for-1
	Promotion	Prominent position of the food or beverages, presence of promoters

**Table 2:** Instruments applied to measure food environments.

Type of Food Environment	n (%)
Store	1425 (73.9)
Restaurant	354 (18.4)
Street food	133 (6.9)
Institution	16 (0.8)
Total	1928 (100)

**Table 3:** Reliability analysis of items for the dimensions of each instrument to measure food environments.

Variables	Institution	Street Food	Store	Restaurant
Healthy food availability				
Fruit	0.76	0.73	0.90	-
Vegetables	0.76	0.71	0.91	-
Dairy products	0.75	0.74	0.88	-
Legumes	-	0.74	0.89	-
Meats and eggs	0.76	0.75	0.88	-
Cereals	0.75	0.76	0.89	-
Beverages	0.72	0.73	0.89	-
Prepared foods	0.75	0.74	-	-
Breakfast	-	-	-	0.66
Lunch	-	-	-	0.61
Teatime	-	-	-	0.64
Dinner	-	-	-	0.62
Unhealthy food availability	0.75	0.68	0.88	0.68
Variety	0.75	0.72	0.86	0.61
Healthy food advertising	0.78	0.71	-	0.69
Total	0.77	0.74	0.90	0.68

**Table 4:** Results of applied guidelines. Pilot study.

Food Group	Range	X ± SD
Store food environment (n = 1425)		
Availability		
Fruit	0–4	1.01 ± 1.07
Vegetables	0–1	0.62 ± 0.48
Dairy products	0–6	2.24 ± 2.14
Legumes	0–1	0.39 ± 0.48
Meats and eggs	0–4	1.54 ± 1.30
Cereals	0–4	0.55 ± 0.95
Beverages	0–5	2.56 ± 1.77
Total healthy food availability	0–28	10.62 ± 7.47
Unhealthy foods	0–13	6.08 ± 4.07
Variety	0–8	2.07 ± 2.10
Healthy food advertising	0–7	0.88 ± 0.90
Restaurant food environment (n = 354)		
Availability		
Breakfast	0–6	0.42 ± 1.05
Lunch	0–5	1.31 ± 1.33
Teatime	0–6	0.60 ± 1.07
Dinner	0–6	1.09 ± 1.25
Total healthy food availability	0–22	3.41 ± 3.34
Unhealthy foods	0–10	3.95 ± 1.75
Variety	0–4	0.89 ± 1.15
Healthy food advertising	0–3	0.05 ± 0.26
Street food environment (n = 133)		
Availability		
Fruit	0–2	0.37 ± 0.54
Vegetables	0–1	0.36 ± 0.48
Dairy products	0–1	0.02 ± 0.15
Legumes	0–1	0.04 ± 0.19
Meats and eggs	0–1	0.11 ± 0.31
Cereals	0–1	0.02 ± 0.12
Beverages	0–4	0.29 ± 0.81
Total healthy food availability	0–7	1.32 ± 1.28
Prepared foods	0–2	0.12 ± 0.35
Unhealthy foods	0–7	1.18 ± 1.56
Variety	0–3	0.36 ± 0.71
Healthy food advertising	0–2	0.74 ± 0.95
Institution food environment (n = 16)		
Availability		
Fruit	0–3	0.88 ± 0.89
Vegetables	0–1	0.19 ± 0.40
Dairy products	0–3	0.63 ± 0.89
Legumes	0–0	-
Meats and eggs	0–1	0.13 ± 0.34
Cereals	0–2	0.50 ± 0.73
Beverages	0–4	2.00 ± 1.32
Total healthy food availability	0–14	4.88 ± 3.69
Prepared foods	0–10	2.25 ± 2.86
Unhealthy foods	0–9	3.38 ± 2.78
Variety	0–4	0.94 ± 1.44
Healthy food advertising	0–3	0.31 ± 0.87

SD: standard deviation.

## Data Availability

Not applicable.

## Supplementary Materials

The following supporting information can be downloaded at: https://www.mdpi.com/article/10.3390/ijerph192113806/s1. Table S1: Food environment “Institution”; Table S2: Food environment “Street food”; Table S3: Food environment “Store”; Table S4: Food environment “Restaurant”.
