# Supplementary material for: Development and Validation of a Set of Instruments to Measure Food Environments
Source: Int J Environ Res Public Health. 2022 Oct 24;19(21):13806. doi: 10.3390/ijerph192113806 (PMC9653673; doi:10.3390/ijerph192113806)
Supplement: Supplementary file 1 [file ijerph-19-13806-s001.zip › ijerph-1888641-supplementary.pdf]

Supplementary Files: Instruments to measure food environments.

Table S1. FOOD ENVIRONMENT: INSTITUTIONAL

**I. AVAILABILITY**<br>**The location sells:**		**YES**	**NO**
**Fruit**	Fruit with no added sugar or non-caloric sweetener (washed, chopped, canned, tetra pack, or frozen).		
	100% fruit juices with no added sugar or non-caloric sweeteners.		
	Dried fruits with no added sugar or salt (sun-dried peaches, raisins, apples, bananas, etc.).		
	Nuts with no added sugar or salt (peanuts, almonds, etc.).		
**Vegetables**	Fresh, canned, tetra pack, or frozen vegetables (including potatoes, corn, sweet potato, etc.).		
**Dairy products**	Low-fat milk (liquid and/or powdered).		
	Flavored milk with no added sugar or no “HIGH IN” labels.		
	Cultured milk with no added sugar or no “HIGH IN” labels.		
	Yogurt with no added sugar or no “HIGH IN” labels.		
	Fresh cheese.		
	Yellow cheese with no “HIGH IN” labels.		
**Legumes**	Beans, lentils, chick peas, peas, and others (dried, canned, tetra pack, or frozen).		
**Meats and eggs**	Fresh or frozen low-fat meats (chicken, turkey, red meats, low-fat cuts such as round roast).		
	Fresh or frozen fish and/or seafood.		
	Ready-to-eat fish (canned and/or tetra pack) low in sodium and water.		
	Fresh or cooked eggs.		
**Cereals**	Breakfast cereals with no added sugar or no “HIGH IN” labels.		
	Cereals (quinoa, amaranth, brown rice, husked wheat, oats, etc.).		
	Whole grain bread (rye, bran, chia, oat, or other).		
**Beverages**	Bottled natural and/or sparkling/light sparkling water.		
	Flavored water with no added sugar or no “HIGH IN” labels.		
	100% fruit juices with no added sugar or no “HIGH IN” labels.		
	Fruit nectar with no added sugar or no “HIGH IN” labels.		
	Beverages with no added sugar or no “HIGH IN” labels.		
**Others**	Vegetable oil (olive, canola, sunflower, etc.).		
	Ice cream with no added sugar or no “HIGH IN” labels.		
	Tea, coffee, and infusions with no added sugar or non-caloric sweetener.		
	**Subtotal Foods**		

**Prepared foods**	Healthy options of vegetable soups or vegetable salads with no sauces or fried foods.		
	Ready-to-eat sandwiches with whole grain bread (with vegetables and/or dairy products, low-fat meats such as fish, turkey, chicken, low-fat red meats, and eggs).		
	Ready-to-eat whole grain tortillas (tacos, burritos) with vegetables and/or dairy products, low-fat meats such as fish, turkey, chicken, low-fat red meats, and eggs.		
	Healthy main dish options (based on legumes, vegetables, fish, seafood and/or algae such as cochayuyo).		
	Healthy dessert options (prepared foods based on fruit and dairy products with no added sugar and low-fat).		
	Tea, coffee, and infusions with no added sugar or non-caloric sweetener.		
	**Subtotal Prepared foods**		

**Unhealthy foods**	Salty snacks with more than one “HIGH IN” label (potato chips and other processed foods such as cheese puffs, and puffs).		
	Sweet snacks with more than one “HIGH IN” label (such as cookies, chocolates, pastries).		
	Sausages and packaged cold cuts with more than one “HIGH IN” label (ham, mortadella, salami, wieners, etc.).		
	Sauces and dressings such mayonnaise, ketchup, and mustard with more one “HIGH IN” label.		
	Sweet sauces (Nutella, ice cream sauces, etc.) with more than one “HIGH IN” label.		
	Ice cream with more than one “HIGH IN” label.		
	Baked or fried sweet dough containing refined sugars (“calzones rotos”: type of fried dough, sopaipillas pasadas: fried dough with squash dipped in dark brown sugar syrup, etc.).		
	Baked or fried salted dough with or without filling (sopaipillas: fried dough, empanadas: type of baked or fried turnover with filling, fried rolls, etc.).		
	Fast food (pizzas, hot dogs, french fries, sandwiches, etc.).		
	Soft drinks, juices, and nectars processed with added sugar and the “HIGH IN” label.		
	Sports or energy drinks.		
	Unhealthy sweet/salty snacks sold in bulk (not packaged).		
	**Subtotal Unhealthy foods**		

**II. VARIETY**<br>**The location has:**		**YES**	**NO**
	Fruit (3 or more).		
	Vegetables (3 or more).		
	Low-fat milk or yogurt (3 or more) with no added sugar or no “HIGH IN” labels.		
	Low-fat cheese and fresh cheese (2 or more) with no “HIGH IN” labels.		
	Fresh, dried, frozen, packaged, canned, and/or tetra pack legumes (2 or more).		
	Low-fat meats (2 or more types).		
	Breakfast cereals (3 or more) with no added sugar or no “HIGH IN” labels.		
	Bottled natural and/or sparkling and flavored water (3 or more) with no added sugar or no “HIGH IN” labels.		
	More than one option of prepared foods with smaller portions (for children).		
	**TOTAL VARIETY**		

III. ADVERTISING<br>**The location has advertising aimed at:**		YES	NO
	Food-based dietary guidelines for the Chilean population (GABA) (increase the consumption of fruit, vegetables, fish, dairy products, and water; decrease the consumption of salt/sodium/sugars etc.).		
	Preference for foods with less labels (Law N°20.606).		
	Decrease in the price of food or prepared foods, healthy prepared foods such as fruit, vegetables, dairy products, legumes, or fish.		
	Prominent placement of healthy foods such as fruit, vegetables, dairy products, legumes, or fish.		
	Discounts, such as 2-for-1, and promotion for the purchase of healthy prepared foods/food and packs.		
	Healthy food products (natural) are located near the checkout counter.		
	Presence of promoters providing information and/or tastings and/or samples of healthy food.		
	**TOTAL ADVERTISING**		

Table S2. FOOD ENVIRONMENT: STREET FOOD

I. AVAILABILITY<br>**The location sells:**		YES	NO
**Fruit**	Fruit with no added sugar or non-caloric sweetener (washed, chopped, canned, tetra pack, or frozen).		
	100% fruit juices with no added sugar or non-caloric sweeteners.		
	Dried fruits with no added sugar or salt (sun-dried peaches, raisins, apples, bananas, etc.).		
	Nuts with no added sugar or salt (peanuts, almonds, etc.).		
**Vegetables**	Fresh, canned, tetra pack, or frozen vegetables (including potatoes, corn, sweet potato, etc.).		
**Dairy products**	Low-fat milk (liquid and/or powdered).		
	Flavored milk with no added sugar or no "HIGH IN" labels.		
	Cultured milk with no added sugar or no “HIGH IN” labels.		
	Yogurt with no added sugar or no “HIGH IN” labels.		
	Fresh cheese.		
	Yellow cheese with no “HIGH IN” labels.		
**Meats and eggs**	Fresh or frozen low-fat meats (chicken, turkey, red meats, low-fat cuts such as round roast).		
	Fresh or frozen fish and/or seafood.		
	Ready-to-eat fish (canned and/or tetra pack) low in sodium and water.		
	Fresh or cooked eggs.		

**Cereals**	Breakfast cereals with no added sugar or no "HIGH IN" labels.		
	Cereals (quinoa, amaranth, brown rice, husked wheat, oats, etc.).		
	Whole grain bread (rye, bran, chia, oat, or other).		
**Beverages**	Bottled natural and/or sparkling/light sparkling water.		
	Flavored water with no added sugar or no "HIGH IN" labels.		
	100% fruit juices with no added sugar or no "HIGH IN" labels.		
	Fruit nectar with no added sugar or no "HIGH IN" labels.		
	Beverages with no added sugar or no "HIGH IN" labels.		
**Others**	Vegetable oil (olive, canola, sunflower, etc.).		
	Ice cream with no added sugar or no "HIGH IN" labels.		
	Tea, coffee, and infusions with no added sugar or non-caloric sweetener.		
	**Subtotal Foods**		

**Prepared foods**	Healthy options of vegetable soups or vegetable salads with no sauces or fried foods.		
	Ready-to-eat sandwiches with whole grain bread (with vegetables and/or dairy products, low-fat meats such as fish, turkey, chicken, low-fat red meats, and eggs).		
	Ready-to-eat whole grain tortillas (tacos, burritos) with vegetables and/or dairy products, low-fat meats such as fish, turkey, chicken, low-fat red meats, and eggs.		
	Healthy main dish options (based on legumes, vegetables, fish, seafood and/or algae such as cochayuyo).		
	Healthy dessert options (prepared foods based on fruit and dairy products with no added sugar and low-fat).		
	Tea, coffee, and infusions with no added sugar or non-caloric sweetener.		
	**Subtotal Prepared foods**		

**Unhealthy foods**	Salty snacks with more than one "HIGH IN" label (potato chips and other processed foods such as cheese puffs, and puffs).		
	Sweet snacks with more than one "HIGH IN" label (such as cookies, chocolates, pastries).		
	Sausages and packaged cold cuts with more than one "HIGH IN" label (ham, mortadella, salami, wieners, etc.).		
	Sauces and dressings such mayonnaise, ketchup, and mustard with more one "HIGH IN" label.		
	Sweet sauces (Nutella, ice cream sauces, etc.) with more than one "HIGH IN" label.		
	Ice cream with more than one "HIGH IN" label.		
	Baked or fried sweet dough containing refined sugars ("calzones rotos": type of fried dough, sopaipillas pasadas: fried dough with squash dipped in dark brown sugar syrup, etc.).		
	Baked or fried salted dough with or without filling (sopaipillas: fried dough, empanadas: type of baked or fried turnover with filling, fried rolls, etc.).		
	Fast food (pizzas, hot dogs, french fries, sandwiches, etc.).		
	Soft drinks, juices, and nectars processed with added sugar and the "HIGH IN" label.		
	Sports or energy drinks.		
	Unhealthy sweet/salty snacks sold in bulk (not packaged).		

	**Subtotal Unhealthy foods**		

**II. VARIETY**<br>**The location has:**		**YES**	**NO**
	Fruit (2 o más).		
	Vegetables (2 o más).		
	Low-fat milk or yogurt (3 or more) with no added sugar or no “HIGH IN” labels.		
	Low-fat cheese and fresh cheese (2 or more) with no “HIGH IN” labels.		
	Low-fat meats (2 or more types).		
	Breakfast cereals with no added sugar or no “HIGH IN” labels.		
	Bottled natural and/or sparkling and flavored water (2 or more) with no added sugar and no “HIGH IN” labels.		
	More than one option of prepared foods with smaller portions (for children).		
	**TOTAL VARIETY**		

**III. ADVERTISING**<br>**The location has advertising aimed at:**		**YES**	**NO**
	Food-based dietary guidelines for the Chilean population (GABA) (increase the consumption of fruit, vegetables, fish, dairy products, and water; decrease the consumption of salt/sodium/sugars etc.).		
	Preference for foods with less labels (Law N°20.606).		
	Decrease in the price of food or prepared foods, healthy prepared foods such as fruit, vegetables, dairy products, legumes, or fish.		
	Prominent placement of healthy foods such as fruit, vegetables, dairy products, legumes, or fish.		
	Discounts, such as 2-for-1, and promotion for the purchase of healthy prepared foods/food and packs.		
	Healthy food products (natural) are located near the checkout counter.		
	Presence of promoters providing information and/or tastings and/or samples of healthy food.		
	**TOTAL ADVERTISING**		

Table S3. FOOD ENVIRONMENT: STORES

**I. AVAILABILITY**<br>**The location sells:**		**YES**	**NO**
**Fruit**	Fruit with no added sugar or non-caloric sweetener (washed, chopped, canned, tetra pack, or frozen).		
	100% fruit juices with no added sugar or non-caloric sweeteners.		
	Dried fruits with no added sugar or salt (sun-dried peaches, raisins, apples, bananas, etc.).		
	Nuts with no added sugar or salt (peanuts, almonds, etc.).		
**Vegetables**	Fresh, canned, tetra pack, or frozen vegetables (including potatoes, corn, sweet potato, etc.).		
**Dairy products**	Low-fat milk (liquid and/or powdered).		
	Flavored milk with no added sugar or no "HIGH IN" labels.		
	Cultured milk with no added sugar or no “HIGH IN” labels.		
	Yogurt with no added sugar or no “HIGH IN” labels.		
	Fresh cheese.		
	Yellow cheese with no “HIGH IN” labels.		
**Legumes**	Beans, lentils, chick peas, peas, and others (dried, canned, tetra pack, or frozen).		
**Meats and eggs**	Fresh or frozen low-fat meats (chicken, turkey, red meats, low-fat cuts such as round roast).		
	Fresh or frozen fish and/or seafood.		
	Ready-to-eat fish (canned and/or tetra pack) low in sodium and water.		
	Fresh or cooked eggs.		
**Cereals**	Breakfast cereals with no added sugar or no “HIGH IN” labels.		
	Cereals (quinoa, amaranth, brown rice, husked wheat, oats, etc.).		
	Whole grain bread (rye, bran, chia, oat, or other).		
**Beverages**	Bottled natural and/or sparkling/light sparkling water.		
	Flavored water with no added sugar or no “HIGH IN” labels.		
	100% fruit juices with no added sugar or no “HIGH IN” labels.		
	Fruit nectar with no added sugar or no “HIGH IN” labels.		
	Beverages with no added sugar or no “HIGH IN” labels.		
**Others**	Vegetable oil (olive, canola, sunflower, etc.).		
	Ice cream with no added sugar or no “HIGH IN” labels.		
	Tea, coffee, and infusions with no added sugar or non-caloric sweetener.		
	**Subtotal Foods**		

**Prepared foods**	Healthy options of vegetable soups or vegetable salads with no sauces or fried foods.		
	Ready-to-eat sandwiches with whole grain bread (with vegetables and/or dairy products, low-fat meats such as fish, turkey, chicken, low-fat red meats, and eggs).		
	Ready-to-eat whole grain tortillas (tacos, burritos) with vegetables and/or dairy products, low-fat meats such as fish, turkey, chicken, low-fat red meats, and eggs.		
	Healthy main dish options (based on legumes, vegetables, fish, seafood and/or algae such as cochayuyo).		
	Healthy dessert options (prepared foods based on fruit and dairy products with no added sugar and low-fat).		
	Tea, coffee, and infusions with no added sugar or non-caloric sweetener.		
	**Subtotal Prepared foods**		

**Unhealthy foods**	Salty snacks with more than one "HIGH IN" label (potato chips and other processed foods such as cheese puffs, and puffs).		
	Sweet snacks with more than one "HIGH IN" label (such as cookies, chocolates, pastries).		
	Sausages and packaged cold cuts with more than one "HIGH IN" label (ham, mortadella, salami, wieners, etc.).		
	Sauces and dressings such mayonnaise, ketchup, and mustard with more one "HIGH IN" label.		
	Sweet sauces (Nutella, ice cream sauces, etc.) with more than one "HIGH IN" label.		
	Ice cream with more than one "HIGH IN" label.		
	Baked or fried sweet dough containing refined sugars ("calzones rotos": type of fried dough, sopaipillas pasadas: fried dough with squash dipped in dark brown sugar syrup, etc.).		
	Baked or fried salted dough with or without filling (sopaipillas: fried dough, empanadas: type of baked or fried turnover with filling, fried rolls, etc.).		
	Fast food (pizzas, hot dogs, french fries, sandwiches, etc.).		
	Soft drinks, juices, and nectars processed with added sugar and the "HIGH IN" label.		
	Sports or energy drinks.		
	Unhealthy sweet/salty snacks sold in bulk (not packaged).		
	**Subtotal Unhealthy foods**		

**II. VARIETY**<br>**The location has:**		**YES**	**NO**
	Fruit (3 or more).		
	Vegetables (3 or more).		
	Low-fat milk or yogurt (3 or more) with no added sugar or no "HIGH IN" labels.		
	Low-fat cheese and fresh cheese (2 or more) with no "HIGH IN" labels.		
	Fresh, dried, frozen, packaged, canned, and/or tetra pack legumes (2 or more).		
	Low-fat meats (2 or more types).		
	Breakfast cereals (3 or more) with no added sugar or no "HIGH IN" labels.		
	Bottled natural and/or sparkling and flavored water (3 or more) with no added sugar or no "HIGH IN" labels.		
	More than one option of prepared foods with smaller portions (for children).		
	**TOTAL VARIETY**		

III. ADVERTISING<br>**The location has advertising aimed at:**		YES	NO
	Food-based dietary guidelines for the Chilean population (GABA) (increase the consumption of fruit, vegetables, fish, dairy products, and water; decrease the consumption of salt/sodium/sugars etc.).		
	Preference for foods with less labels (Law N°20.606).		
	Decrease in the price of food or prepared foods, healthy prepared foods such as fruit, vegetables, dairy products, legumes, or fish.		
	Prominent placement of healthy foods such as fruit, vegetables, dairy products, legumes, or fish.		
	Discounts, such as 2-for-1, and promotion for the purchase of healthy prepared foods/food and packs.		
	Healthy food products (natural) are located near the checkout counter.		
	Presence of promoters providing information and/or tastings and/or samples of healthy food.		
	**TOTAL ADVERTISING**		

Table S4. FOOD ENVIRONMENT: RESTAURANT

**I. AVAILABILITY**<br>**The location sells:**		YES	NO
**Breakfast**	Fruit with no added sugar or non-caloric sweetener.		
	100% fruit juices with no added sugar or non-caloric sweeteners.		
	Cereals with no added sugar.		
	Dried fruits with no added sugar or salt (sun-dried peaches, raisins, apples, bananas, etc.).		
	Nuts with no added sugar or salt (peanuts, almonds, etc.).		
	Low-fat dairy products with no added sugar or no “HIGH IN” labels.		
	Tea, coffee, and infusions with no added sugar or non-caloric sweetener.		
	Whole grain bread (rye, bran, chia, oat, or other).		
	Healthy spreads for bread such as eggs, avocado, diet jams, and fat-free ham.		
	Sweet doughs, cakes, and tarts with low added sugar.		
	**Subtotal Breakfast**		
**Lunch**	Low-fat and low-sugar appetizers (whole-grain bread, nuts, crackers with fresh cheese, and non-fried vegetables).		
	Healthy entrée options (vegetable soups or vegetable salads with no sauces or fried foods).		
	Healthy main dish options (based on legumes, vegetables, fish, seafood and/or algae such as cochayuyo) not combined with cured meats such as sausages or cold cuts.		
	Healthy dessert options (prepared foods based on fruit and dairy products with no added sugar and low-fat).		
	The menu includes healthy beverage options (water, fruit and vegetable-based juices, and light or no sugar added drinks).		

	**Subtotal Lunch**		
**Tea time**	Fruit with no added sugar or non-caloric sweetener.		
	100% fruit juices with no added sugar or non-caloric sweeteners.		
	Cereals with no added sugar.		
	Dried fruits with no added sugar or salt (sun-dried peaches, raisins, apples, bananas, etc.).		
	Nuts with no added sugar or salt (peanuts, almonds, etc.).		
	Low-fat dairy products with no added sugar or no "HIGH IN" labels.		
	Tea, coffee, and infusions with no added sugar or non-caloric sweetener.		
	Whole grain bread (rye, bran, chia, oat, or other).		
	Healthy spreads for bread such as eggs, avocado, diet jams, and fat-free ham.		
	Sweet doughs, cakes, and tarts with low added sugar.		
	**Subtotal Tea time**		
**Dinner**	Low-fat and low-sugar appetizers (whole-grain bread, nuts, crackers with fresh cheese, and non-fried vegetables).		
	Healthy entrée options (vegetable soups or vegetable salads with no sauces or fried foods).		
	Healthy main dish options (based on legumes, vegetables, fish, seafood and/or algae such as cochayuyo) not combined with cured meats such as sausages or cold cuts.		
	Healthy dessert options (prepared foods based on fruit and dairy products with no added sugar and low-fat).		
	The menu includes healthy beverage options (water, fruit and vegetable-based juices, and light or no sugar added drinks).		
	**Subtotal Dinner**		

**Unhealthy foods**	Salty snacks with more than one "HIGH IN" label (potato chips and other processed foods such as cheese puffs, and puffs).		
	Sweet snacks with more than one "HIGH IN" label (such as cookies, chocolates, pastries).		
	Sausages and packaged cold cuts with more than one "HIGH IN" label (ham, mortadella, salami, wieners, etc.).		
	Sauces and dressings such mayonnaise, ketchup, and mustard with more one "HIGH IN" label.		
	Sweet sauces (Nutella, ice cream sauces, etc.) with more than one "HIGH IN" label.		
	Ice cream with more than one "HIGH IN" label.		
	Baked or fried sweet dough containing refined sugars ("calzones rotos": type of fried dough, sopaipillas pasadas: fried dough with squash dipped in dark brown sugar syrup, etc.).		
	Baked or fried salted dough with or without filling (sopaipillas: fried dough, empanadas: type of baked or fried turnover with filling, fried rolls, etc.).		
	Fast food (pizzas, hot dogs, french fries, sandwiches, etc.).		
	Soft drinks, juices, and nectars processed with added sugar and the "HIGH IN" label.		
	Sports or energy drinks.		
	Unhealthy sweet/salty snacks sold in bulk (not packaged).		
	**Subtotal Unhealthy foods**		
	**TOTAL AVAILABILITY**		

**II. VARIETY**<br>**The location has:**		**YES**	**NO**
	Fruit (3 or more).		
	Vegetables (3 or more).		
	Low-fat milk or yogurt (3 or more) with no added sugar or no “HIGH IN” labels.		
	Low-fat cheese and fresh cheese (2 or more) with no “HIGH IN” labels.		
	Fresh, dried, frozen, packaged, canned, and/or tetra pack legumes (2 or more).		
	Low-fat meats (2 or more types).		
	At least one breakfast cereal with no added sugar or no “HIGH IN” labels.		
	Bottled natural and/or sparkling and flavored water (2 or more) with no added sugar and no “HIGH IN” labels.		
	More than one option of prepared foods with smaller portions (for children).		
	**TOTAL VARIETY**		

**III. ADVERTISING**<br>**The location has advertising aimed at:**		**YES**	**NO**
	Food-based dietary guidelines for the Chilean population (GABA) (increase the consumption of fruit, vegetables, fish, dairy products, and water; decrease the consumption of salt/sodium/sugars etc.).		
	Preference for foods with less labels (Law N°20.606).		
	Decrease in the price of food or prepared foods, healthy prepared foods such as fruit, vegetables, dairy products, legumes, or fish.		
	Prominent placement of healthy foods such as fruit, vegetables, dairy products, legumes, or fish.		
	Discounts, such as 2-for-1, and promotion for the purchase of healthy prepared foods/food and packs.		
	Healthy food products (natural) are located near the checkout counter.		
	**TOTAL ADVERTISING**		
